# A distinct serum metabolic profile characterizes osteosarcopenia: identifying potential metabolic biomarkers

**DOI:** 10.3389/fendo.2026.1865132

**Published:** 2026-08-14

**Authors:** Hui Gao, Huihui Wu, Liang Guo, Zhisheng Zhang, Jiangang Chen, Haihong Chen, Yiding Zhao, Zhi Wang

**Affiliations:** 1 School of Gongli Hospital Medical Technology, University of Shanghai for Science and Technology, Shanghai, China; 2 Department of Orthopedics I, Gongli Hospital of Shanghai Pudong New Area, Shanghai, China; 3 Artificial Intelligence and Omics Research Center, AigenX Bioscience Co., Ltd., Shanghai, China

**Keywords:** discovery-level biomarker candidates, LC–MS, osteoporosis, osteosarcopenia, sarcopenia, serum metabolomics

## Abstract

**Background:**

Osteosarcopenia, defined by the coexistence of sarcopenia and osteoporosis, is increasingly recognized as a high-risk geriatric musculoskeletal syndrome. Although it is commonly diagnosed by applying separate criteria for sarcopenia and osteoporosis, whether osteosarcopenia has a distinct systemic metabolic phenotype remains unclear. This study aimed to characterize the serum metabolomic profile of osteosarcopenia and to identify discovery-level candidate metabolites that may distinguish osteosarcopenia from osteoporosis alone.

**Methods:**

We conducted a cross-sectional untargeted metabolomics study using high-resolution liquid chromatography–mass spectrometry in 104 older adults, including healthy controls (HC, n = 30), participants with osteoporosis only (OP, n = 30), sarcopenia only (IS, n = 14), and osteosarcopenia (OS, n = 30). Because the IS group was relatively small, comparisons involving IS were treated as exploratory and hypothesis-generating. The primary mechanistic and biomarker analyses focused on the OP versus OS comparison. Differential metabolites, enriched pathways, and preliminary discriminatory performance were assessed using multivariate modeling, false discovery rate correction, pathway enrichment analysis, covariate-adjusted regression, and receiver operating characteristic analysis. Age, sex, body mass index, and estimated glomerular filtration rate were included as covariates.

**Results:**

Osteosarcopenia showed a distinct serum metabolomic profile, particularly when compared with osteoporosis alone. In the OP versus OS comparison, differential metabolites were mainly involved in amino acid metabolism, energy metabolism, purine metabolism, and membrane lipid remodeling. Key alterations included reduced branched-chain amino acids, decreased hypoxanthine, increased creatine, and changes in sphingomyelin and phosphatidylcholine species. Pathway enrichment analysis highlighted valine, leucine, and isoleucine degradation; arginine and proline metabolism; and purine metabolism as significantly enriched pathways. Among the discovery-level candidate metabolites, hypoxanthine and leucine showed apparent discriminatory potential for distinguishing OS from OP, with area under the curve values of 0.87 and 0.79, respectively. These values should be interpreted as discovery-set estimates rather than externally validated diagnostic performance.

**Conclusions:**

Osteosarcopenia was associated with a multi-pathway serum metabolic signature that differed from osteoporosis alone, supporting the possibility that osteosarcopenia represents an integrated musculoskeletal phenotype rather than merely the additive coexistence of muscle and bone loss. The identified metabolites provide preliminary mechanistic insight and discovery-level biomarker candidates, but they require targeted quantitative validation and external cohort confirmation before clinical application.

**Clinical trial registration:**

## 1 Introduction

Osteosarcopenia (OS) – also known as the concurrent muscle–bone reduction syndrome – refers to the combined presence of both sarcopenia and osteoporosis, typically in older individuals (1). Sarcopenia is a progressive, generalized loss of skeletal muscle mass, strength, and physical performance, whereas osteoporosis is characterized by low bone mass and microarchitectural deterioration of bone tissue, leading to reduced bone strength and increased fracture risk (2, 3). When these conditions co-occur, patients experience significantly elevated risks of adverse outcomes, including frailty, falls, fractures, hospitalizations, and mortality, along with impaired mobility and a marked decline in quality of life (4, 5). Epidemiologically, OS is increasingly recognized as a serious global health issue in aging populations (1). Prevalence estimates vary widely depending on the diagnostic criteria and population studied, ranging roughly from 5% to 37% among community-dwelling older adults (1). A recent meta-analysis reported an overall global osteosarcopenia prevalence of about 18.5% among older adults, with rates rising to nearly 25% in octogenarians (6). Given that the worldwide population of people over 60 is projected to double from ~841 million in 2013 to ~2 billion by 2050, the absolute number of individuals affected by osteosarcopenia will increase dramatically (1). This trend portends a substantial growing burden of frailty and fractures in the coming decades, firmly positioning OS as an emerging public health challenge on a global scale (7, 8).

Despite the significant clinical impact of OS, it is still diagnosed in practice using an additive operational framework that a patient meets the independent diagnostic criteria for both sarcopenia and osteoporosis (9–11). This approach fails to account for the biological features that distinguish OS as an integrated muscle–bone phenotype (12). Such a conceptual and diagnostic limitation contributes to difficulties in the early identification of OS, obscures disease-specific mechanisms, and a lack of targeted intervention strategies (13). Therefore, it is critical to clarify the fundamental differences between OS and isolated musculoskeletal conditions—particularly osteoporosis alone and to characterize the distinct pathophysiological features of OS, in order to overcome current challenges in its diagnosis and treatment.

Notably, the homeostatic regulation of muscle and bone does not occur in isolation (14). Instead, these two tissues are tightly interconnected through various molecular signaling pathways and local cytokine crosstalk (15). Both muscle and bone have endocrine functions: they secrete cytokines and growth factors (myokines and osteokines, respectively) that influence each other’s metabolic homeostasis (16, 17). During aging, there is evidence of a “synergistic deterioration” of muscle and bone. As muscle mass and strength decline, bone loss is accelerated; conversely, decreasing bone mass can promote muscle atrophy and functional impairment, jointly exacerbating the progression of OS (18). This coordinated muscle–bone degeneration is accompanied by systemic metabolic reprogramming, suggesting that elucidating the metabolic characteristics of OS will be key to uncovering its unique pathogenic mechanisms (19).

Metabolomics provides a powerful systems-level approach to capture dynamic changes in small-molecule metabolites, which represent the endpoints of physiological and pathological processes (19). This technique enables the discovery of disease-specific metabolic fingerprints and the identification of disrupted metabolic pathways, making it an ideal method to investigate the metabolic regulatory mechanisms underlying OS (20). Previous metabolomic studies focused on sarcopenia only or isolated osteoporosis have revealed metabolic disturbances such as chronic low-grade inflammation and imbalances in energy metabolism, suggesting that these two conditions may share a common metabolic basis (21–25). However, a critical question remains unanswered: when muscle and bone deteriorate simultaneously, do they induce a unique systemic metabolic reprogramming that produces a metabolic phenotype distinct from either condition alone? Addressing this issue would not only provide key molecular evidence supporting OS as a distinct disease entity, but also facilitate the development of OS-specific diagnostic biomarkers and targeted therapeutic strategies (26).

To address these gaps, the present study conducted a high-resolution untargeted metabolomic analysis on serum samples from a cohort comprising four groups: healthy controls (HC), sarcopenia only (IS), isolated osteoporosis (OP), and osteosarcopenia (OS). Our objectives were to (1) systematically compare the overall metabolomic profiles of the four groups in order to define the core metabolic features of OS; (2) identify the metabolites that are uniquely altered in OS compared to the other groups and analyze the key enriched metabolic pathways associated with these metabolites; and (3) perform a preliminary evaluation of the diagnostic performance of these characteristic metabolites in distinguishing OS from isolated osteoporosis. Because the IS group had a smaller sample size, all IS-related analyses were considered exploratory and hypothesis-generating. The main mechanistic and biomarker analyses were therefore centered on the OP vs OS comparison. Through this metabolomics-based approach, we aim to provide new insights into the status of OS as an independent disease entity and its underlying pathophysiology, recognizing that candidate biomarkers identified in this discovery cohort require targeted and external validation before clinical translation.

## 2 Methods

### 2.1 Study design and population

This cross-sectional study aimed to compare serum metabolic profiles among older adults with varying musculoskeletal health statuses. This was an observational, cross-sectional metabolomics study. Participants underwent an additional venous blood draw solely for research sample collection; however, no therapeutic, behavioral, or diagnostic intervention was prospectively assigned, and no intervention effect was evaluated. The protocol was approved by the Ethics Committee of Gongli Hospital of Shanghai Pudong New Area, Shanghai, China), and written informed consent was obtained from all participants.

We enrolled adults aged ≥55 years. Exclusion criteria were: psychiatric/cognitive impairment or sensory deficits hindering communication; severe endocrine, nutritional, or metabolic diseases; long-term use of bone metabolism-affecting medications (e.g., corticosteroids, thyroid hormones); implanted electronic devices interfering with body composition measurements; active malignancy or major organ failure; uncontrolled peripheral vascular disease or neuropathy; history of specific musculoskeletal disorders (e.g., osteoarthritis, rheumatoid arthritis); major acute medical events within the prior 3 months; or chronic kidney disease (eGFR < 60 mL/min/1.73 m²). All enrolled participants had an eGFR ≥ 60.

Bone mineral density (BMD) was measured by dual-energy X-ray absorptiometry (DXA). Osteoporosis (OP) was diagnosed per WHO criteria (T-score ≤ –2.5 at lumbar spine or femoral neck). Sarcopenia was diagnosed according to the EWGSOP2 operational criteria. Low handgrip strength was defined as <27 kg for men and <16 kg for women, which prompted assessment of appendicular skeletal muscle mass via bioelectrical impedance analysis (BIA). The skeletal muscle index (SMI) was calculated as appendicular skeletal muscle mass divided by height squared, with low muscle mass defined as SMI <7.0 kg/m² for men and <5.5 kg/m² for women.

Based on these assessments, participants were classified into four groups: osteosarcopenia (OS, concurrent sarcopenia and OP), osteoporosis only (OP), sarcopenia only (IS), and healthy controls (HC). Baseline data were collected. As an exploratory metabolomic study without prior effect size estimates, no formal sample size calculation was performed. We enrolled all eligible participants during the study period to maximize cohort size and phenotypic representation.

### 2.2 Sample collection, processing, and metabolomic profiling

Fasting venous blood samples (2–5 mL) were collected in the morning into plain tubes after an overnight fast. Samples were allowed to clot and then centrifuged to isolate serum. After discarding hemolyzed samples, 300 µL aliquots were stored at –80 °C. For analysis, samples were thawed on ice. Metabolite extraction was performed on an automated Starlid™ workstation. Briefly, 100 µL of serum was added to 400 µL of 80% aqueous methanol, vortexed, incubated on ice for 5 min, and centrifuged (15,000 g, 20 min, 4 °C). The supernatant was diluted with water to a final methanol concentration of 53%, centrifuged again under identical conditions, and the final supernatant was collected for LC-MS analysis. LC-MS analysis was conducted by AigenX Biosciences Co., Ltd. (Shanghai, China). Chromatographic separation was performed on a Vanquish UPLC system equipped with a Waters ACQUITY UPLC BEH Amide column (2.1 × 50 mm, 1.7 µm), using a water/acetonitrile gradient. Mass spectrometric detection was performed on an Orbitrap Exploris 120 instrument in both positive and negative ionization modes. Raw data were converted to mzXML format using ProteoWizard. Metabolites were identified by matching against the AigenXDB (v3.0) in-house library via a proprietary R-based pipeline and quantified using CD 3.3 software. Metabolite annotations were classified according to confidence levels. Level 1 annotations were confirmed by matching with authentic standards using retention time (RT), MS1, and MS2 information; Level 2 annotations were matched with public spectral databases using MS1 and MS2 information; Level 3 annotations were assigned based on theoretical library matching with predicted RT, MS1, and MS2 information; and Level 4 features were regarded as unknown compounds. In the present dataset, 483 metabolites were annotated at Level 1, 1047 at Level 2, and 86 at Level 3. Representative candidate metabolites highlighted or discussed in this study were annotated at Level 1 or Level 2, and detailed annotation information is provided in **Supplementary Table 6**. To improve transparency and reproducibility of metabolite identification, **Supplementary Table 6** provides annotation information for these representative metabolites, including MS2-matched metabolite name, MS2 matching score, annotation level, m/z, retention time, molecular formula, ionization mode, and available HMDB, KEGG, and PubChem identifiers.

### 2.3 Data preprocessing and quality control

Raw data were processed using XCMS for peak picking, alignment, and correction. Features with >50% missing values were removed; remaining missing values were imputed via a K-nearest neighbors algorithm. Data were normalized by median centering and unit variance scaling. Quality control (QC) samples, prepared from a pooled mixture of all study samples, were analyzed at regular intervals (every 10 study samples) throughout the analytical sequence, accounting for approximately 10% of total injections. System stability was assessed by calculating the relative standard deviation (RSD) of peak intensities across all QC samples; only features with RSD < 30% were retained for further analysis. System stability was confirmed by principal component analysis (PCA) of quality control (QC) samples, which showed tight clustering (**Supplementary Figure S2**).

### 2.4 Statistical and enrichment analysis

Group comparisons for clinical variables used ANOVA or Kruskal-Wallis test (continuous) and chi-square or Fisher’s exact test (categorical).

Differential metabolite candidates were first screened by combining multivariate and univariate criteria, using an OPLS-DA variable importance in projection (VIP) score >1.0 and a raw P-value <0.05. To account for multiple testing across all detected metabolites and control the false discovery rate (FDR), raw P-values from univariate tests were subsequently adjusted using the Benjamini–Hochberg procedure. Metabolites with FDR-adjusted P-values (q values) <0.05 were considered statistically significant and were prioritized for representative metabolite interpretation, pathway enrichment analysis, covariate-adjusted assessment, and downstream biological discussion.

Significant metabolites were mapped to the KEGG database for pathway enrichment analysis, and enrichment significance was assessed using a hypergeometric test. Pathways with an enrichment FDR < 0.05 were considered significant and visualized in bubble plots (bubble size: metabolite count; color: -log_10_(FDR)). A pathway interaction network was constructed using the ggraph and tidygraph R packages, with edges weighted by the Jaccard similarity coefficient of shared metabolites. To account for potential confounders, covariate-adjusted linear regression models were applied to selected representative metabolites in the primary OP versus OS comparison, with metabolite abundance as the dependent variable and group status as the main independent variable. Age, sex, BMI, and eGFR were included as covariates.

### 2.5 Diagnostic model evaluation

The diagnostic potential of key metabolites for distinguishing OP from OS was evaluated using receiver operating characteristic (ROC) curve analysis. The area under the curve (AUC) was calculated for individual metabolites. A multiple logistic regression model incorporating the three selected metabolites, hypoxanthine, leucine, and sarcosine, was then built, and its predicted probabilities were used to generate the ROC curve for the combined panel. Given the absence of an independent external validation cohort, all AUC values were interpreted as apparent discovery-set estimates. No independent external validation or internal resampling-based optimism correction was performed; therefore, all ROC results should be interpreted as preliminary discriminatory performance rather than validated diagnostic accuracy. These analyses were intended to evaluate preliminary biomarker potential rather than to establish a clinically deployable diagnostic model. These analyses were performed using R (v4.5.1) and GraphPad Prism (v10.1.2).

## 3 Results

### 3.1 Characteristics of the study population

The baseline characteristics of the study participants are summarized in **Table 1**. Age and estimated glomerular filtration rate (eGFR) were comparable across the four groups, with no statistically significant differences observed (P > 0.05). In contrast, body mass index (BMI), grip strength, skeletal muscle index (SMI), and bone mineral density showed significant group-wise differences (P < 0.05), with the osteosarcopenia (OS) group exhibiting the most unfavorable musculoskeletal profile. Covariate-adjusted analyses of selected representative metabolites in the primary OP versus OS comparison were performed with age, sex, BMI, and eGFR included as covariates.

**Table 1:** Baseline characteristics of the study participants.

Characteristic	HC (n = 30)	IS (n = 14)	OP (n = 30)	OS (n = 30)
Age (years)	64.5 ± 8.2^a^	67.0 ± 6.8^a^	68.2 ± 8.5^a^	69.5 ± 9.6^a^
Sex, female, n (%)	19 (63.3) ^a^	9 (64.3) ^a^	20 (66.7) ^a^	21 (70.0) ^a^
Fracture history, n (%)	0 (0.0)^a^	0 (0.0)^a^	8 (26.7)^b^	12 (40.0)^c^
eGFR (mL/min/1.73 m^2^)	85.5 ± 8.2^a^	84.0 ± 8.5^a^	84.4 ± 8.8^a^	83.0 ± 9.1^a^
BMI (kg/m^2^)	24.1 ± 2.0^a^	21.8 ± 1.9^b^	23.5 ± 2.3^a^	20.9 ± 1.9^b^
Grip Strength (kg)	31.5 ± 4.1^a^	24.8 ± 3.5^b^	28.1 ± 5.2^a^	22.3 ± 3.8^c^
Femoral Neck BMD (g/cm^2^)	0.95 ± 0.12^a^	0.91 ± 0.10^a^	0.68 ± 0.08^b^	0.62 ± 0.07^b^
Lumbar Spine BMD (g/cm^2^)	1.05 ± 0.14^a^	1.01 ± 0.13^a^	0.78 ± 0.09^b^	0.72 ± 0.08^b^
SMI (kg/m^2^)	7.5 ± 0.8^a^	6.0 ± 0.7^b^	7.0 ± 0.8^a^	5.8 ± 0.7^b^

Continuous variables are presented as mean ± SD and were compared among groups using one-way ANOVA. Categorical variables are presented as n (%) and were compared using chi-square or Fisher’s exact tests, as appropriate. *Post hoc* pairwise comparisons were performed using Tukey’s HSD test for continuous variables and chi-square or Fisher’s exact tests with Bonferroni correction for categorical variables, as appropriate. Within each row, groups not sharing a common superscript letter differ significantly at P < 0.05. HC, healthy controls; IS, sarcopenia only; OP, osteoporosis only; OS, osteosarcopenia; BMI, body mass index; BMD, bone mineral density; eGFR, estimated glomerular filtration rate; SMI, skeletal muscle index.

### 3.2 Distinct serum metabolic profiles revealed by OPLS-DA

**Figure 1:**
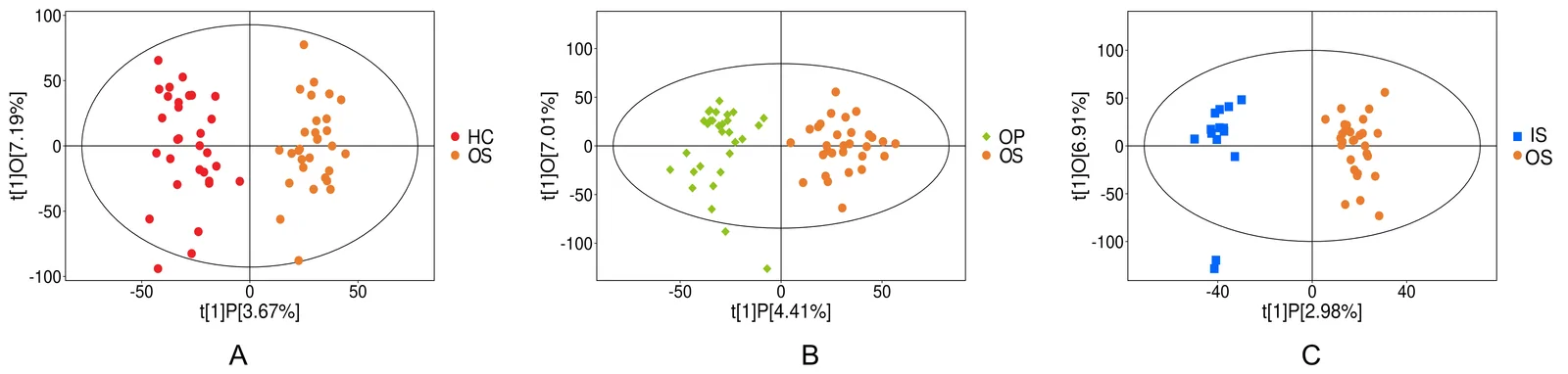
OPLS-DA score plots differentiating metabolic profiles between groups. HC, healthy controls group; IS, sarcopenia only group; OP, osteoporosis only group; OS, osteosarcopenia group. Each point represents one sample. **(A)** OPLS-DA score plot for HC versus OS. **(B)** OPLS-DA score plot for OP versus OS. **(C)** OPLS-DA score plot for IS versus OS. Panel **(C)** represents an exploratory comparison because of the limited sample size of the IS group (n = 14) and should not be interpreted with the same statistical weight as the HC versus OS or OP versus OS comparisons.

Initial assessment of the serum metabolome supported analytical stability, as evidenced by the tight clustering of quality control samples (**Supplementary Figure S2**). We then applied supervised orthogonal partial least squares-discriminant analysis (OPLS-DA) to further delineate intergroup metabolic differences. As shown in **Figure 1**, the OS group showed separation from the HC group (**Figure 1A**). Importantly, the OS group also showed a distinct metabolic profile compared with the OP group (**Figure 1B**), suggesting that OS may have metabolic features not solely attributable to osteoporosis alone. Furthermore, although based on a limited sample size, the OS group exhibited a separation trend from the IS group in the OPLS-DA score plot (**Figure 1C**), which was interpreted only as an exploratory, hypothesis-generating comparison. Permutation testing was performed for the OPLS-DA models (200 permutations; permutation P < 0.01 for all comparisons; **Supplementary Figure S3**, **Supplementary Table 2**), supporting model stability while recognizing the exploratory nature of the IS-related comparison.

### 3.3 Identification of differential metabolites and an OS-associated metabolic signature

**Figure 2:**
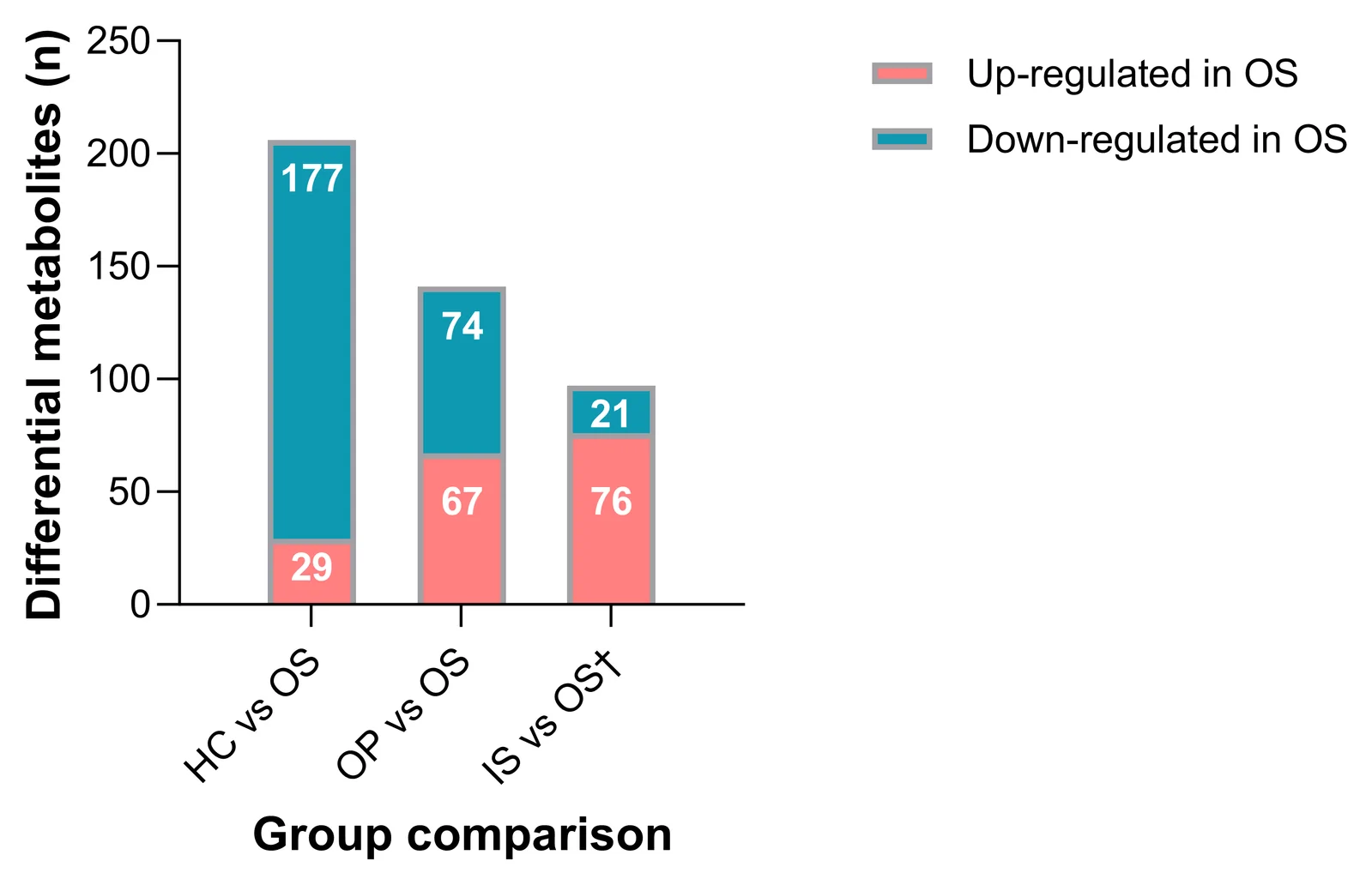
Overview of the number of screening-level differential metabolite candidates across group comparisons. Stacked bars show the numbers of up-regulated and down-regulated metabolite candidates in OS relative to each comparison group. Screening-level differential metabolite candidates were defined as those with VIP >1.0 and raw P-value <0.05. † The IS vs OS comparison was considered exploratory because of the limited sample size of the IS group (n = 14); therefore, the number of metabolite candidates in this comparison should be interpreted descriptively rather than as a definitive estimate. HC, healthy controls; IS, sarcopenia only; OP, osteoporosis only; OS, osteosarcopenia.

To delineate the landscape of metabolic alterations, we performed systematic pairwise comparisons (**Figure 2**). Initial screening identified a substantial number of screening-level differential metabolite candidates in all comparisons. The HC versus OS contrast showed the highest total number of candidates (206), which were predominantly downregulated (177 downregulated vs 29 upregulated). The OP versus OS comparison yielded 141 candidates with a more balanced distribution between upregulated (67) and downregulated (74) species. While the IS versus OS comparison also indicated 97 candidates, this result should be interpreted descriptively because of the smaller sample size of the IS group, and this comparison was considered exploratory.

To provide a more intuitive visualization of metabolite contributions to the primary OP versus OS discrimination, we added a VIP plot of selected biologically interpretable metabolites ranked by VIP score (**Supplementary Figure S4**). Detailed statistical metrics for the representative metabolites highlighted in the main text are provided in **Supplementary Table 3**.

**Figure 3:**
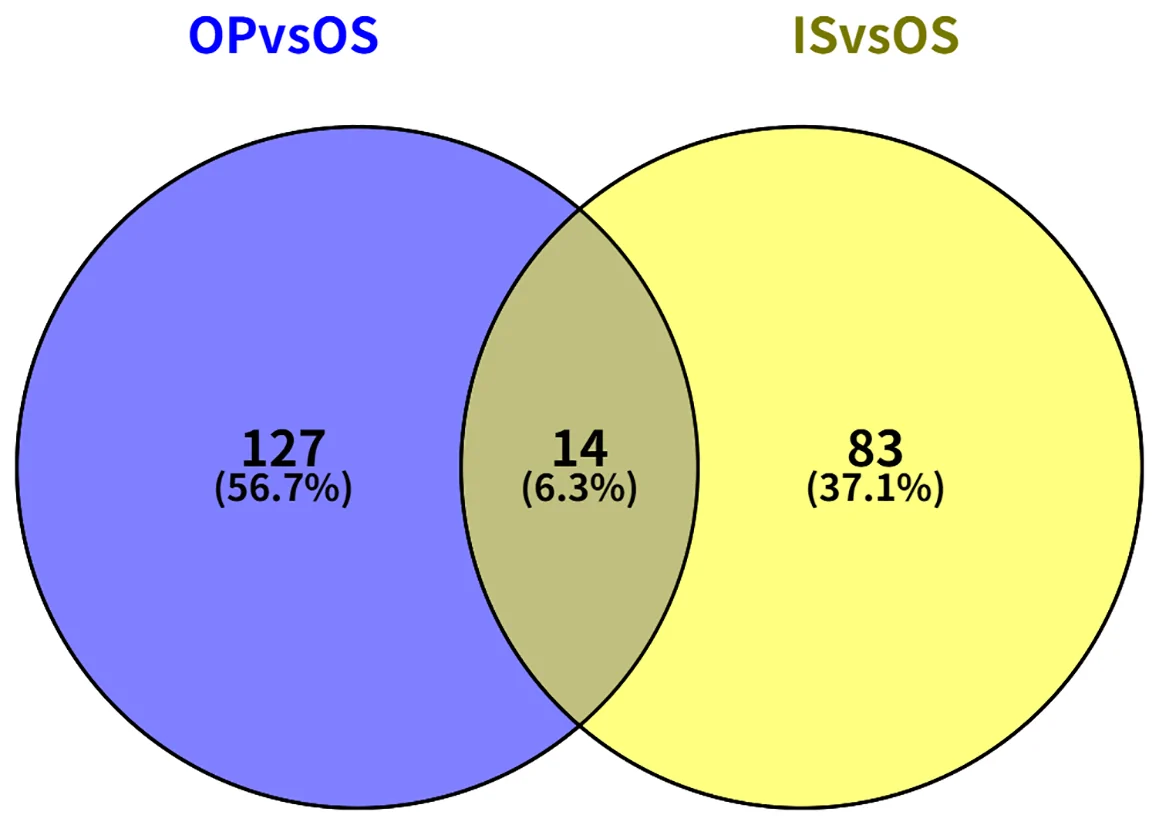
Exploratory analysis of metabolite overlap between osteosarcopenia and sarcopenia only. The Venn diagram shows the overlap of screening-level differential metabolite candidates identified in the OS versus OP and OS versus IS comparisons using VIP >1.0 and raw P-value <0.05. The intersection contains 14 candidates. The OS versus IS comparison is exploratory because of the limited sample size of the IS group (n = 14), and this overlap should be interpreted as a hypothesis-generating observation for future validation. IS, sarcopenia only; OP, osteoporosis only; OS, osteosarcopenia.

We then performed an exploratory analysis to examine the metabolic relationship between OS and IS. A Venn diagram analysis (**Figure 3**) revealed an overlap of 14 screening-level differential metabolite candidates between the OS versus OP and OS versus IS comparisons. After excluding seven exogenous metabolites (listed in **Supplementary Note 1**), seven endogenous metabolites—histidinol, lactate, N6-methyllysine, Car (18:0), 3-aminopentanoic acid, SM (d34:1), and heptadecanoyl carnitine—were found to be altered in OS patients relative to both OP and IS patients. Given the exploratory nature and limited sample size of the IS group (n = 14), this finding should be regarded as preliminary and hypothesis-generating. Consequently, all subsequent mechanistic and diagnostic evaluations in this report focused on the more balanced OP versus OS comparison.

### 3.4 Key metabolic alterations in osteosarcopenia

To delineate key metabolic alterations in osteosarcopenia (OS), we focused on representative metabolites altered in OS patients relative to those with osteoporosis alone (OP), while also showing their group-level distributions across healthy controls (HC), sarcopenia only (IS), OP, and OS. Representative metabolites were selected from FDR-significant metabolites in the primary OP versus OS comparison (VIP > 1.0 and FDR-adjusted P < 0.05), based on their biological relevance to musculoskeletal-related pathways and their interpretable group-level patterns across the four cohorts. The set included markers of branched-chain amino acid metabolism (leucine, downregulated; 3-methylglutaconic acid, upregulated), energy-related metabolism (creatine, upregulated; hypoxanthine, downregulated), and lipid metabolism (arachidonoyl-L-carnitine, downregulated; SM(d34:1), upregulated). Notably, SM(d34:1) was also identified in the exploratory overlap analysis (**Figure 3**). Detailed statistical metrics (VIP, raw P-value, FDR-adjusted P-value, and fold change) for these and additional representative metabolites in the primary OP versus OS comparison are provided in **Supplementary Table 3**. Additional box plots of representative metabolites are provided in **Supplementary Figure S5**. Covariate-adjusted linear regression analyses restricted to the OP and OS groups showed that most selected representative metabolites remained nominally significant after adjustment for age, sex, BMI, and eGFR, whereas pyruvate was attenuated after adjustment (**Supplementary Table 4**).

**Figure 4:**
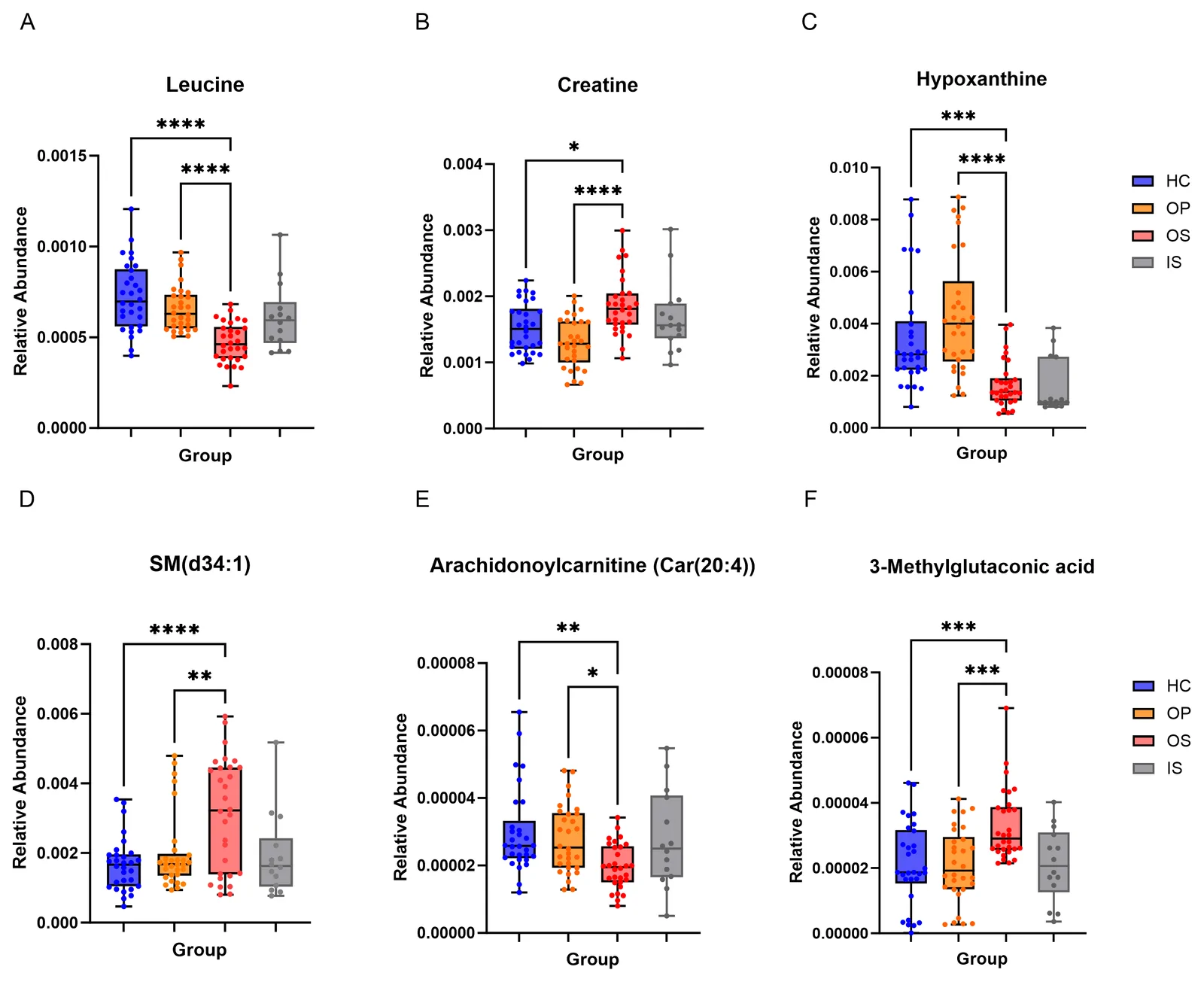
Comparison of key metabolite levels across the four study groups. Box plots display the relative abundance of six key metabolites across the four groups: **(A)** leucine, **(B)** creatine, **(C)** hypoxanthine, **(D)** SM (d34:1), **(E)** arachidonoylcarnitine, and **(F)** 3-methylglutaconic acid. Intergroup comparisons were performed using the Kruskal–Wallis test followed by Dunn’s *post hoc* test. Asterisks indicate significant intergroup differences: *P < 0.05, **P < 0.01, ***P < 0.001. HC, healthy controls; IS, sarcopenia only; OP, osteoporosis only; OS, osteosarcopenia.

Serum levels across the four cohorts (HC, IS, OP, OS) showed group-level differences in these representative metabolites (**Figure 4**). The OS group showed lower levels of leucine and hypoxanthine, elevated creatine, and shifts in specific lipid-related metabolites. Because this was a cross-sectional study, these patterns should be interpreted as group-level metabolic differences rather than evidence of progressive metabolic dysfunction or an advanced disease state. Covariate-adjusted linear regression analyses restricted to the OP and OS groups showed that all representative metabolites listed in **Supplementary Table 4**, except pyruvate, remained nominally significant after adjustment for age, sex, BMI, and eGFR.

### 3.5 Pathway enrichment analysis of OS versus OP differences

**Figure 5:**
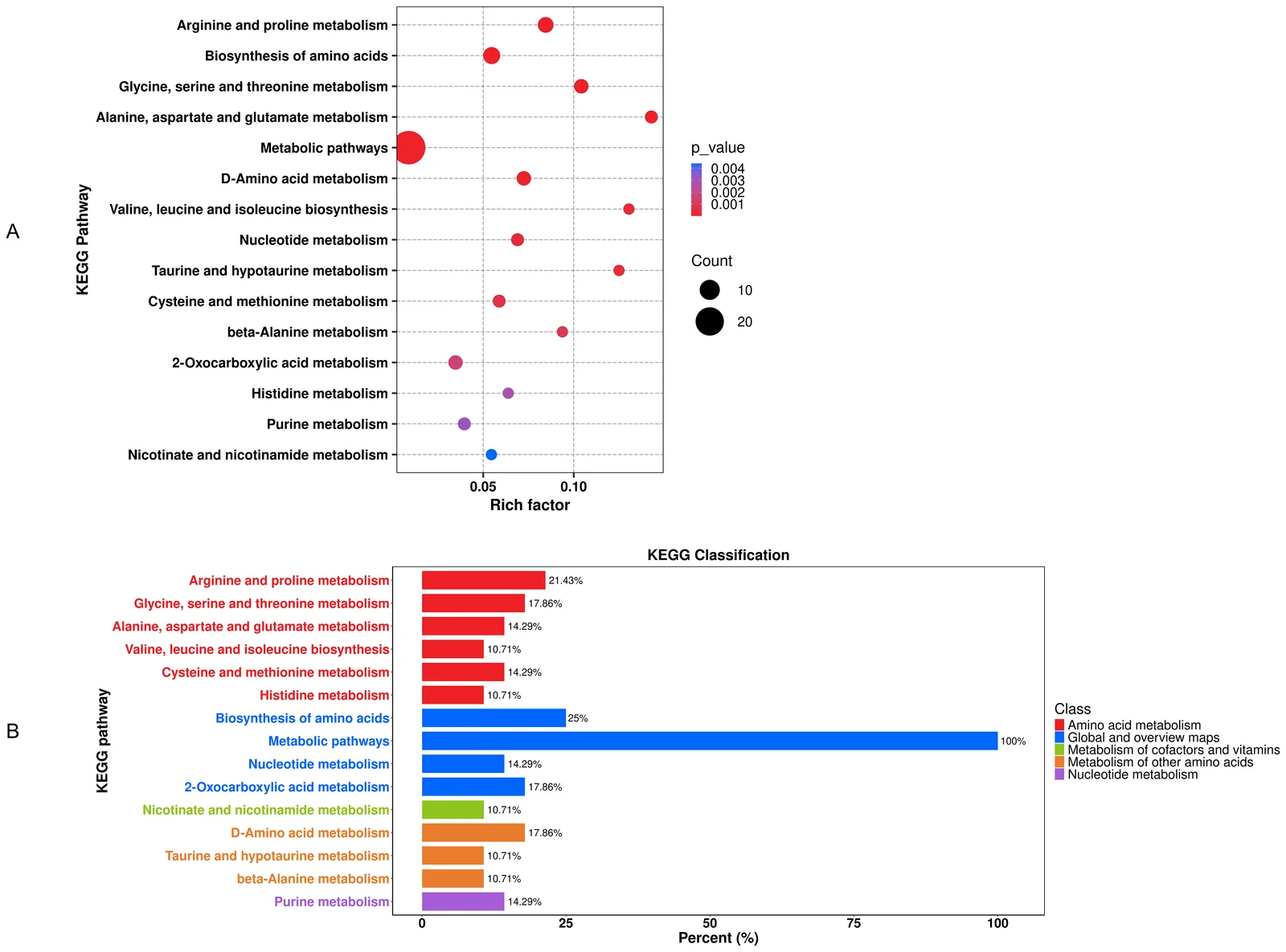
Enriched KEGG pathways for metabolites differentially expressed between the osteoporosis-only (OP) and osteosarcopenia (OS) groups. **(A)** Bubble plot of significantly enriched pathways (FDR-adjusted P < 0.05). Bubble size corresponds to the number of differentially expressed metabolites mapped to each pathway, and color intensity represents enrichment significance. The rich factor is the ratio of the number of differential metabolites to the total number of metabolites annotated in that pathway. **(B)** Bar chart showing the percentage of differential metabolites mapped to each significantly enriched pathway, categorized by KEGG superclass. OP, osteoporosis only; OS, osteosarcopenia.

KEGG pathway enrichment analysis of the differentially expressed metabolites between OP and OS (FDR < 0.05) revealed significant alterations in several metabolic processes (**Figure 5**). The most notably enriched pathways included valine, leucine and isoleucine degradation, arginine and proline metabolism, and purine metabolism. These results suggest that OS is associated with coordinated alterations in metabolic pathways related to amino acid metabolism, energy-related metabolism, and nitrogen metabolism when compared with osteoporosis alone.

### 3.6 Integrated metabolic network analysis

**Figure 6:**
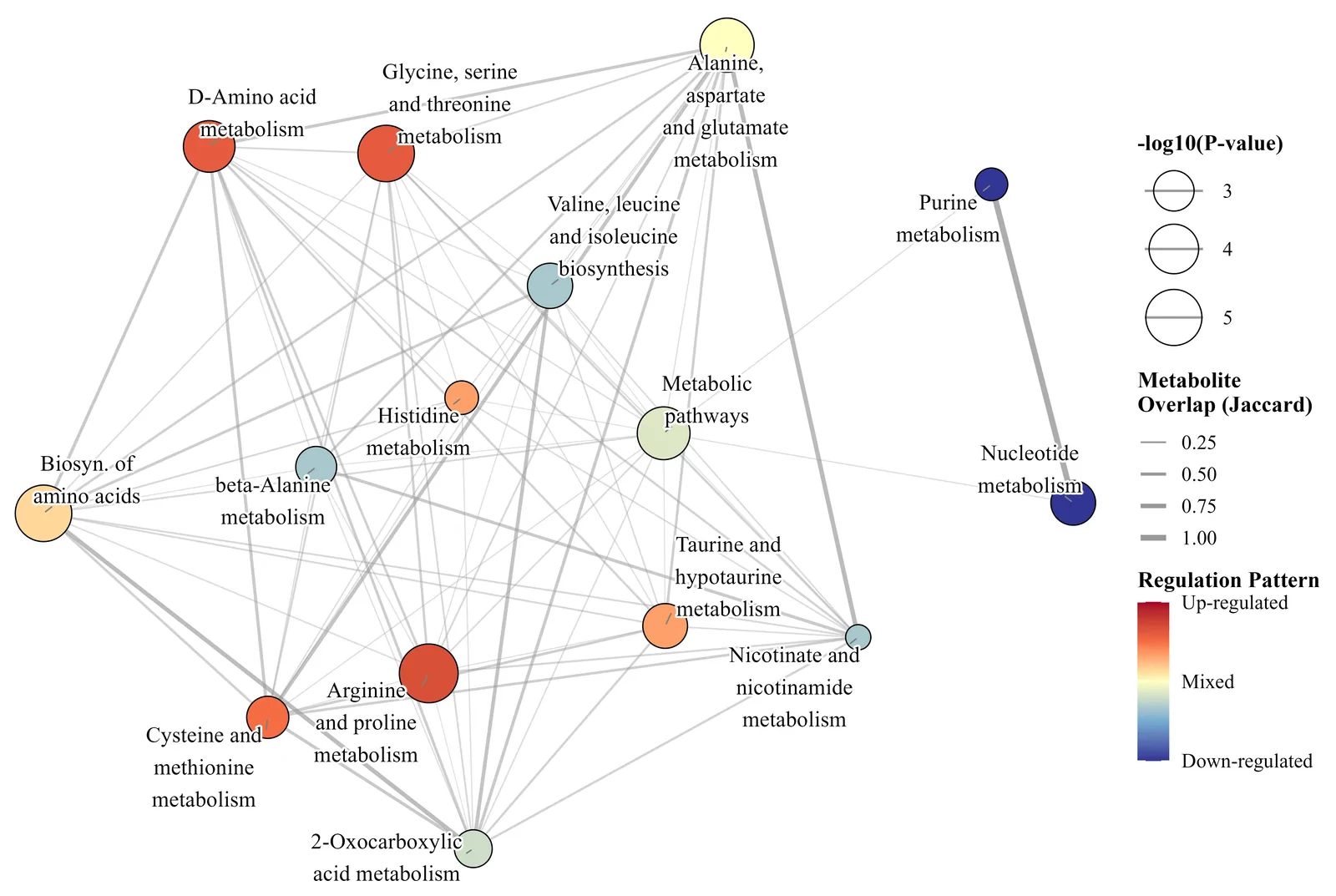
Interaction network of enriched metabolic pathways.

An interaction network of the top 15 enriched pathways (FDR < 0.05) delineated three interconnected metabolic axes altered in OS (**Figure 6**): (1) energy- and creatine-related metabolism, (2) amino acid and collagen-related metabolism, and (3) cellular protection and redox homeostasis. Central hub pathways, such as “biosynthesis of amino acids” exhibited high connectivity. This network pattern supports the presence of coordinated pathway-level metabolic alterations in OS, but it should be interpreted as a hypothesis-generating framework rather than direct evidence of causal pathophysiological mechanisms.

Nodes represent significantly enriched KEGG pathways (FDR-adjusted P < 0.05), sized by -log_10_(P-value) and colored by regulation direction (red: up, blue: down). Edge thickness indicates pathway similarity (Jaccard coefficient). The clustering of amino acid metabolism pathways suggests coordinated pathway-level alterations in the OP versus OS comparison.

### 3.7 Preliminary discriminatory performance of key metabolites (ROC analysis)

**Figure 7:**
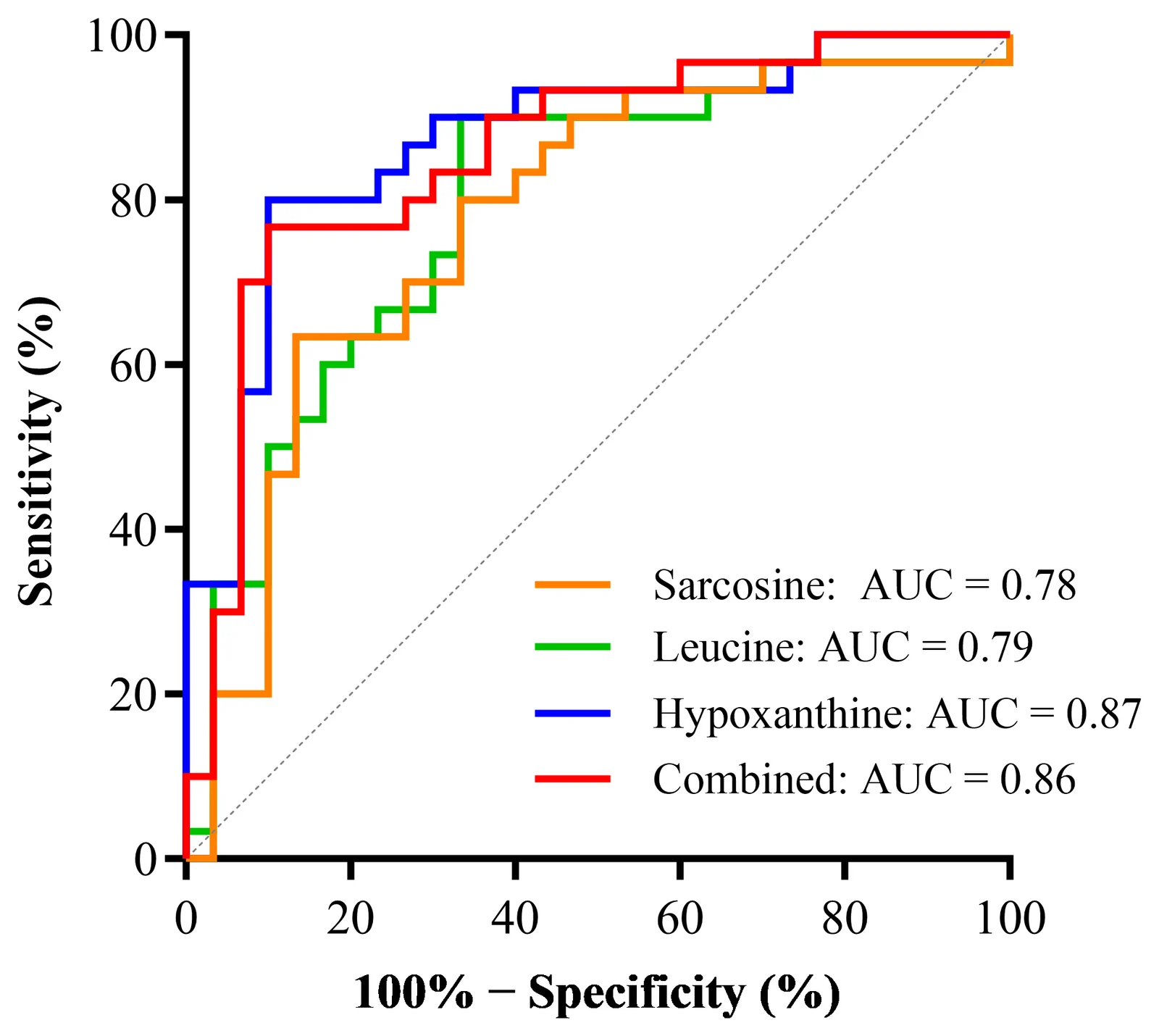
Receiver operating characteristic curves for sarcosine, leucine, hypoxanthine, and the combined three-metabolite model for distinguishing OP from OS. AUC values were calculated in the discovery cohort and were not externally validated. OP, osteoporosis only; OS, osteosarcopenia.

Finally, to evaluate the preliminary biomarker potential of key metabolites, we performed receiver operating characteristic (ROC) analysis. For distinguishing OS from OP—the primary focus of our diagnostic evaluation—hypoxanthine showed the highest apparent discriminatory performance (AUC = 0.87, 95% CI: 0.78–0.96), followed by leucine (AUC = 0.79) and sarcosine (AUC = 0.78). A logistic regression model combining these three metabolites yielded an AUC of 0.86 (**Figure 7**). Because the combined model did not outperform hypoxanthine alone, we did not interpret the panel as superior to the best single metabolite. All ROC-derived AUC values were obtained from the discovery dataset and should therefore be interpreted as preliminary discovery-set estimates rather than externally validated diagnostic performance. In the comparison of OS versus HC, isovalerylcarnitine also showed preliminary discriminatory potential (AUC = 0.82). The complete ROC analysis results, including specificity and sensitivity for individual metabolites and the combined model, are provided in **Supplementary Table 5**.

## 4 Discussion

### 4.1 The metabolic landscape of osteosarcopenia: toward a pathophysiological integration

Our untargeted serum metabolomic analysis identified a metabolic profile associated with osteosarcopenia (OS) that was distinguishable from osteoporosis alone. Supervised OPLS-DA modeling showed group discrimination, with the most informative and statistically balanced contrast observed between OS and OP. This comparison yielded 141 screening-level differential metabolite candidates and therefore served as the primary basis for subsequent representative metabolite and pathway-level interpretation of the osteosarcopenic metabolic phenotype.

Exploratory cross-comparison with the IS group provided additional contextual information. The modest overlap of metabolites altered in OS relative to both OP and IS was consistent with the possibility that OS is not merely the additive coexistence of sarcopenia and osteoporosis. However, because the IS group was underpowered, these overlapping metabolites should be interpreted as hypothesis-generating signals that may reflect metabolic changes arising from concurrent muscle and bone impairment.

Based on these findings, we conceptualize the OS-associated metabolic phenotype as comprising three interrelated components: metabolic alterations shared with sarcopenia-related dysfunction, alterations linked to osteoporosis-related remodeling, and additional changes that may arise from impaired muscle–bone metabolic crosstalk. These components support the view that OS may represent an integrated musculoskeletal phenotype with metabolic features that differ from isolated osteoporosis.

**Figure 8:**
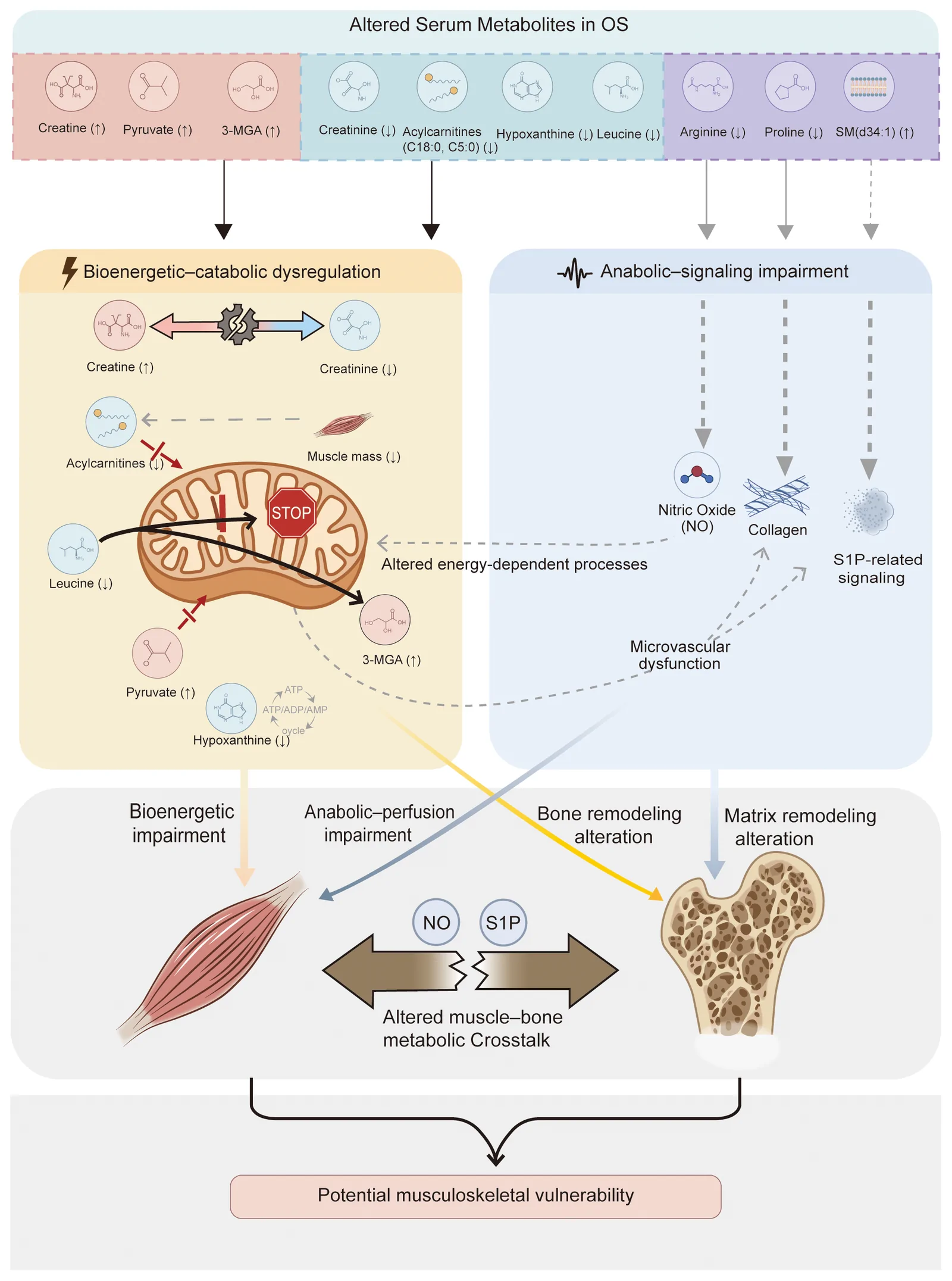
Proposed working model of systemic metabolic dysregulation in osteosarcopenia.

To integrate these observations, we developed a working model of systemic metabolic dysregulation in OS (**Figure 8**). In this model, alterations in amino acid metabolism, bioenergetic pathways, and lipid-mediated signaling may converge to impair both muscle function and bone remodeling. Rather than establishing causality, this model provides a conceptual framework for interpreting the observed metabolomic changes and for guiding future targeted and longitudinal studies.

This schematic integrates the main metabolomic findings into a hypothesis-generating framework. Arrows indicate the observed direction of change in serum metabolites and selected musculoskeletal phenotypes in OS. These alterations are organized into two interacting domains: bioenergetic and catabolic dysregulation, and anabolic and signaling support impairment. Together, these domains may contribute to altered muscle–bone metabolic crosstalk involving putative signaling mediators such as nitric oxide and sphingosine-1-phosphate. Solid arrows indicate proposed metabolic relationships, whereas dashed lines represent putative attenuated processes. Abbreviations: 3-MGA, 3-methylglutaconic acid; S1P, sphingosine-1-phosphate; NO, nitric oxide.

Figure created with Adobe Illustrator.

### 4.2 Amino acid metabolism: BCAA depletion and altered amino acid handling in osteosarcopenia

As illustrated in the working model shown in **Figure 8**, amino acid metabolism represented one of the major altered metabolic domains in OS. In particular, serum leucine and isoleucine levels were lower in the OS group, suggesting reduced circulating availability of branched-chain amino acids (BCAAs). Because leucine is an important nutrient signal involved in the regulation of mammalian target of rapamycin complex 1 (mTORC1)-mediated muscle protein synthesis, reduced leucine availability may be relevant to impaired muscle anabolic signaling in OS (27). However, this interpretation should be made cautiously, as circulating BCAA levels can be influenced by dietary intake, protein turnover, physical activity, and systemic metabolic status.

In parallel with lower BCAA levels, 3-methylglutaconic acid (3-MGA), a metabolite linked to leucine catabolism and mitochondrial metabolic processes, was increased in OS. The coexistence of reduced leucine and elevated 3-MGA may indicate altered BCAA handling and incomplete metabolic processing rather than providing direct evidence of a blocked mitochondrial BCAA degradation pathway. This pattern is consistent with prior evidence linking BCAA metabolism to musculoskeletal aging (28), but the present findings should be regarded as associative and hypothesis-generating. Further targeted metabolomic and functional studies are needed to determine whether these changes reflect altered dietary substrate availability, increased amino acid utilization, impaired mitochondrial catabolism, or a combination of these processes.

Alanine was also increased in OS, contrasting with the reduction in BCAAs. As alanine participates in the glucose–alanine cycle and can serve as a gluconeogenic substrate, its elevation may reflect adaptive amino acid redistribution under conditions of metabolic stress. Together, the reduced BCAA levels, increased 3-MGA, and elevated alanine suggest that OS is associated with disturbed amino acid homeostasis. These alterations may either contribute to or reflect impaired muscle protein balance and systemic metabolic stress; however, causal relationships cannot be inferred from the present cross-sectional metabolomic data.

Overall, the amino acid profile of OS points to a coordinated disturbance involving BCAA availability, amino acid catabolism, and metabolic adaptation. These findings provide a biochemical context for understanding muscle-related metabolic vulnerability in OS and support the need for future studies integrating dietary assessment, targeted amino acid quantification, and longitudinal measures of muscle and bone outcomes.

### 4.3 Energy-related metabolic alterations in osteosarcopenia

Energy-related metabolic alterations represented another prominent feature of the OS-associated serum metabolomic profile. In the present study, OS was characterized by changes in metabolites related to the creatine–creatinine axis, glycolytic intermediates, acylcarnitine metabolism, and purine metabolism. These findings suggest that OS may involve broad disturbances in energy-related metabolic pathways linked to cellular energy buffering and substrate utilization. However, because of the cross-sectional design, whether these changes represent causes, consequences, or compensatory responses cannot be determined.

Creatine and creatinine showed divergent patterns in OS. Serum creatine was increased, whereas creatinine, the end product of creatine metabolism, was reduced (29). Because the phosphocreatine system contributes to cellular energy buffering, elevated creatine may reflect altered creatine turnover or adaptive changes in energy-related metabolism (30). In contrast, lower creatinine may be partly related to reduced muscle mass, as creatinine production has been associated with muscle status and muscle cross-sectional area (31). However, serum creatine and creatinine can also be influenced by renal handling, diet, muscle mass, and supplement use. Therefore, the divergent creatine–creatinine pattern should be interpreted as an energy-related metabolic signal rather than direct evidence of failed energy compensation.

Pyruvate was also altered in OS in the primary differential metabolite analysis, suggesting possible changes in glycolytic metabolism or mitochondrial substrate handling. Because pyruvate links glycolysis to mitochondrial oxidative metabolism, its accumulation may reflect altered glucose-derived energy metabolism (32). However, this difference was attenuated after adjustment for age, sex, BMI, and eGFR, indicating that the pyruvate signal should be interpreted as a pathway-level exploratory finding rather than an independently robust metabolite difference. Nevertheless, serum pyruvate alone cannot establish a specific mitochondrial defect, and future studies incorporating targeted metabolomics, lactate/pyruvate ratios, and direct mitochondrial function measures are needed to clarify its biological significance.

Acylcarnitine-related changes further indicated altered substrate utilization in OS. Several acylcarnitine species were reduced, which differs from the elevation of acylcarnitines often observed in incomplete fatty acid β-oxidation in other metabolic disorders (33). One possible explanation is that reduced acylcarnitines may reflect limited fatty acid mobilization, altered carnitine shuttle activity, or reduced formation of acylcarnitine intermediates. This interpretation is consistent with evidence linking lower muscle carnitine acyltransferase activity to frailty (34), and reduced acylcarnitine species have also been reported in sarcopenic populations (35). In particular, reduced isovalerylcarnitine, a leucine-derived acylcarnitine, may be related to altered BCAA catabolism. The reduction in long-chain acylcarnitines, including stearoylcarnitine, may also be relevant to mitochondrial stress and lipid substrate metabolism (36, 37). However, these serum changes should not be taken as direct evidence of impaired fatty acid oxidation without targeted flux or tissue-level validation.

Purine metabolism also appeared to differ between OS and OP. Hypoxanthine, a purine metabolite involved in ATP salvage pathways, was lower in OS than in OP (38). In the OP group, relatively higher hypoxanthine may reflect preserved or different purine turnover under metabolic stress (39). By contrast, reduced hypoxanthine in OS may indicate altered purine salvage capacity or increased downstream utilization. However, hypoxanthine is sensitive to multiple factors, including diet, renal function, tissue ischemia, and sample handling; therefore, its interpretation as an energy-related marker should remain cautious and requires targeted validation.

Collectively, these findings suggest that OS is associated with coordinated alterations in several energy-related metabolic pathways, including creatine metabolism, glycolytic intermediates, acylcarnitine metabolism, and purine metabolism. Rather than demonstrating a definitive energy failure, the present data support a hypothesis that OS involves altered systemic substrate handling and energy-related metabolic adaptation. These changes may be biologically relevant to both muscle dysfunction and bone remodeling, but their causal role requires confirmation in longitudinal studies with targeted quantitative assays and functional measurements.

### 4.4 Potential muscle–bone crosstalk pathways: arginine–proline metabolism and lipid remodeling

The right panel of **Figure 8** highlights metabolic pathways that may be relevant to muscle–bone communication in OS, particularly arginine–proline metabolism and lipid remodeling. Rather than demonstrating direct disruption of musculoskeletal crosstalk, the present serum metabolomic findings provide hypothesis-generating evidence that these pathways may contribute to the integrated muscle–bone phenotype of OS.

Arginine–proline metabolism is biologically relevant to both muscle and bone. Arginine serves as a substrate for nitric oxide synthase (NOS), which produces nitric oxide (NO), a signaling molecule involved in vascular function, skeletal muscle perfusion, and bone vascular regulation during remodeling (40). Proline is a major component of collagen and is therefore relevant to extracellular matrix formation, bone matrix quality, and connective tissue integrity (41). Alterations in this pathway may therefore reflect changes in metabolic support for both perfusion-related signaling and collagen-associated structural remodeling. However, because this study measured circulating metabolites rather than tissue-level pathway activity, the observed changes should be interpreted as indirect evidence requiring further validation.

Lipid remodeling may represent another metabolic domain linking muscle and bone in OS. In the present study, sphingomyelin species and phosphatidylcholine-related metabolites were altered in OS. Sphingomyelin is a membrane lipid and a precursor of bioactive sphingolipid mediators, including sphingosine-1-phosphate (S1P), which has been implicated in inflammation, tissue repair, and bone remodeling (42). Changes in sphingomyelin metabolism may therefore be relevant to lipid-mediated signaling in the musculoskeletal system. Similarly, altered phosphatidylcholine species may reflect changes in membrane composition, lipid turnover, or inflammatory-metabolic status, processes that have been linked to bone integrity and muscle function in aging populations (43, 44).

Taken together, the alterations in arginine–proline metabolism and lipid-related metabolites suggest that OS may involve impaired metabolic support for vascular signaling, extracellular matrix remodeling, and membrane-associated signaling. These findings support the concept that OS is associated with coordinated metabolic changes across muscle- and bone-relevant pathways. Nevertheless, direct evidence for altered muscle–bone crosstalk will require targeted lipidomics, tissue-level validation, and experimental studies of myokine, osteokine, NO, and sphingolipid signaling.

### 4.5 Diagnostic potential and discovery-level value of metabolic biomarkers

The metabolic differences observed in OS may provide preliminary insight into potential biomarker candidates; however, these findings should be interpreted as discovery-level evidence rather than clinically validated diagnostic performance. ROC analysis of selected metabolites yielded encouraging but preliminary results. For example, leucine and hypoxanthine showed apparent discriminatory potential for distinguishing OS from OP in the discovery dataset (AUCs of 0.79 and 0.87, respectively), consistent with their relevance to muscle anabolism and energy-related metabolism. Because these AUC values were derived from the discovery dataset without external validation or optimism correction, they may be affected by optimism bias and should not be interpreted as established diagnostic accuracy.

Additionally, medium- and short-chain acylcarnitines (e.g., isovalerylcarnitine) demonstrated potential for distinguishing OS from healthy individuals (AUC ≈ 0.82), suggesting their possible relevance as exploratory risk-related markers. Importantly, the combined model including hypoxanthine, leucine, and sarcosine did not outperform hypoxanthine alone; therefore, this panel should not be presented as superior to the best single metabolite. This result may reflect the limited sample size, partial correlation among candidate metabolites, and limited incremental information provided by leucine and sarcosine beyond hypoxanthine.

Given that several single metabolites showed preliminary discriminatory potential, future studies may evaluate panels combining complementary metabolites from disturbed pathways—such as a BCAA-related metabolite, an energy-related purine metabolite, and a lipid remodeling marker. However, such panels require targeted quantitative validation, independent external cohorts, and adjustment for key clinical and lifestyle confounders before clinical translation. In future work, integrating metabolic markers with traditional clinical indicators, including muscle mass, bone mineral density, grip strength, age, sex, renal function, dietary intake, medication use, and supplement exposure, may help construct more robust multidimensional risk models (45). At this stage, the present findings should be regarded as a basis for future biomarker validation rather than as a clinically deployable diagnostic tool.

## 5 Limitations of the study and future directions

This study has several limitations. First, its cross-sectional design precludes causal inference regarding whether the observed metabolic alterations are drivers, consequences, or compensatory changes in osteosarcopenia. Second, although the main findings were based on the relatively balanced OP vs OS comparison, the sarcopenia-only group was small (IS, n = 14); therefore, IS-related analyses should be interpreted as exploratory and hypothesis-generating. Third, although fasting blood sampling minimized acute postprandial effects, detailed habitual dietary intake, medication history, and supplement use were not systematically recorded, which may influence metabolites such as leucine, hypoxanthine, creatine, and lipid-related species. Fourth, although several central candidate metabolites, including leucine, hypoxanthine, creatine, sarcosine, pyruvate, and arachidonoylcarnitine, were annotated at Level 1, some lipid- or pathway-related features, such as SM(d34:1) and 3-methylglutaconic acid, were annotated at Level 2. In addition, the proprietary AigenXDB library may limit full independent reproduction of the annotation workflow. Finally, the candidate metabolites were derived from untargeted metabolomics and were not validated by targeted quantitative assays or independent external cohorts. Therefore, the reported AUC values should be interpreted as apparent discovery-set estimates rather than validated diagnostic performance. Future studies should include larger independent cohorts, standardized dietary, medication, and supplement assessments, targeted LC-MS/MS validation, internal and external validation of diagnostic models, and longitudinal designs to clarify the temporal relationship between metabolic alterations and the development of osteosarcopenia.

## 6 Conclusion

This untargeted metabolomics study suggests that osteosarcopenia (OS) is associated with a distinct serum metabolic profile, supporting its interpretation as an integrated musculoskeletal phenotype beyond the simple co-occurrence of sarcopenia and osteoporosis. We identified multi-pathway differences, most notably in amino acid metabolism, energy-related metabolism, purine metabolism, and membrane lipid remodeling. These findings collectively suggest a state of systemic metabolic disturbance that may be relevant to the parallel deterioration of muscle and bone.

Several metabolites, especially hypoxanthine and leucine, showed preliminary discriminatory potential for distinguishing OS from osteoporosis alone in the discovery dataset. However, these metabolites should be regarded as discovery-level candidates rather than clinically validated diagnostic biomarkers. These findings provide candidate metabolic signals for future targeted validation and may help guide mechanistic studies of OS. Future research should validate these metabolites in larger, longitudinal, and independent cohorts and evaluate whether they add incremental value to established clinical indicators such as bone mineral density, muscle mass, grip strength, age, sex, renal function, diet, medication use, and supplement exposure.

## Data Availability

The raw data supporting the conclusions of this article will be made available by the authors, without undue reservation.
