# Supplementary material for: A distinct serum metabolic profile characterizes osteosarcopenia: identifying potential metabolic biomarkers
Source: Front Endocrinol (Lausanne). 2026 Aug 14;17:1865132. doi: 10.3389/fendo.2026.1865132 (PMC13521881; doi:10.3389/fendo.2026.1865132)
Supplement: Supplementary file 1 [file DataSheet1.docx]

**Supplementary Materials**

**Supplementary Figure S1. Flowchart of participant screening, exclusion, and group allocation.**

Abbreviations: eGFR, estimated glomerular filtration rate; DXA, dual-energy X-ray absorptiometry; EWGSOP2, European Working Group on Sarcopenia in Older People 2; BIA, bioelectrical impedance analysis; SMI, skeletal muscle mass index; HC, healthy control; OP, osteoporosis alone; IS, isolated sarcopenia; OS, osteosarcopenia.

The flowchart details the process from initial screening of potential participants (N = 202) to the final inclusion of the study cohort (N = 104). Eligibility was assessed based on key inclusion criteria (eGFR ≥ 60 mL/min/1.73 m²) and exclusion criteria (detailed in the boxes). All enrolled participants underwent comprehensive assessment of bone mineral density (via DXA) and sarcopenia (via handgrip strength and BIA-derived SMI, following the EWGSOP2 algorithm with Asian cut-offs). The final four comparison groups were defined as shown.

| Characteristic | Enrolled Participants (n = 104) | Eligible Non-Participants (n = 13) | P-value |
| --- | --- | --- | --- |
| Age (years), mean ± SD | 67.3 ± 8.8 | 68.3 ± 6.2 | 0.69 |
| Female, n (%) | 69 (66.3%) | 8 (61.5%) | 0.73 |
| BMI (kg/m²), mean ± SD | 22.6 ± 2.5 | 22.3 ± 2.3 | 0.70 |
| eGFR, mean ± SD | 84.2 ± 8.7 | 84.0 ± 8.0 | 0.94 |

**Supplementary Table S1. Comparison of baseline characteristics between enrolled participants and eligible non-participants.**

Data are presented as mean ± standard deviation (SD) for continuous variables and number (%) for categorical variables. P-values were derived from independent samples t-test (for age, BMI, eGFR) and Chi-square test (for sex).

Abbreviations: SD, standard deviation; BMI, body mass index; eGFR, estimated glomerular filtration rate.

No statistically significant differences were observed in any of the compared baseline characteristics (all P > 0.05), suggesting no major selection bias between the analyzed cohort and the broader eligible population.

**Supplementary Figure S2. Quality control assessment of the LC-MS dataset.**

(A) Injection-order stability plot showing scores on the first principal component (PC1) plotted against the analytical sequence for all samples. Individual study samples (blue) and pooled QC samples (green) are displayed. The horizontal dashed lines indicate the ±2 SD (inner) and ±3 SD (outer) control limits calculated from the PC1 distribution of the QC samples. The tight clustering of QC samples around zero and largely within the ±2 SD limits throughout the run demonstrates minimal analytical drift and excellent instrument stability.

(B) Unsupervised PCA score plot (PC1 vs. PC2) illustrating the clustering of all study samples (blue) and the pooled QC samples (green). The ellipse represents the 95% confidence region for the QC cluster. The tight grouping of QC samples in the center of the PCA space reflects high technical reproducibility and consistent data quality across the analytical batch. In contrast, the broader dispersion of study samples along the first two principal components captures the underlying biological variation among subjects. The compact QC cluster confirms that data normalization and QC-based monitoring effectively controlled technical variability, supporting the robustness of the metabolomic dataset.

**Supplementary Figure S3. Permutation test validation of OPLS-DA models.**

Permutation testing (n = 200) was performed for each OPLS-DA model to assess significance and overfitting risk by comparing the true model’s goodness-of-fit (R²Y) and predictability (Q²) against distributions obtained from randomly permuted class labels.

(A) HC vs OS comparison.

(B) OP vs OS comparison.

(C) IS vs OS comparison.

In each panel, histograms show the frequency distributions of R²Y (blue) and Q² (red) from the permuted models. The vertical dashed lines mark the actual R²Y and Q² values of the original model. In all cases, the true model’s metrics lie far outside the permuted distributions (permutation p < 0.01), supporting that the observed separations were unlikely to be due solely to chance or overfitting.

**Supplementary Table S2 summarizes the key validation metrics for all pairwise OPLS-DA models.**

| Comparison | R²Y (cum) | Q² (cum) | Permutation p (Q²) | Q² intercept | Permutation-supported? |
| --- | --- | --- | --- | --- | --- |
| HC vs OS | 0.94 | 0.409 | <0.01 | −0.70 | Yes |
| OP vs OS | 0.87 | 0.372 | <0.01 | −0.91 | Yes |
| IS vs OS | 0.91 | 0.198 | <0.01 | −0.37 | Yes |
| HC vs OP | 0.86 | 0.340 | <0.01 | −0.68 | Yes |
| HC vs IS | 0.91 | 0.400 | <0.01 | −0.53 | Yes |

Model support in this table refers to permutation-based support against random class-label assignment and does not imply adequate statistical power, particularly for IS-related comparisons, which were considered exploratory because of the limited IS sample size.

Abbreviations: HC, healthy controls; IS, sarcopenia only; OP, osteoporosis only; OS, osteosarcopenia. HC vs OS: Group HC versus OS; OP vs OS: Group OP versus OS; IS vs OS: Group IS versus OS

****Supplementary Figure S4.** **Variable importance in projection (VIP) scores of selected biologically interpretable metabolites in the OP versus OS comparison.** Metabolites are ranked according to VIP scores derived from the OPLS-DA model. The dashed vertical line indicates the VIP threshold of 1.0. Color indicates the direction of change in OS relative to OP. Abbreviations: OP, osteoporosis only; OS, osteosarcopenia.**

**Supplementary Figure S5. Comparison of additional representative metabolite levels across the four study groups.** Box plots display the relative abundance of four additional representative metabolites across the four groups: (A) sarcosine, (B) pyruvate, (C) SM(d18:1/17:0), and (D) proline. Intergroup comparisons were performed using the Kruskal–Wallis test followed by Dunn’s post hoc test. Asterisks indicate significant intergroup differences: *P < 0.05, **P < 0.01, ***P < 0.001, ****P < 0.0001; ns, not significant. Abbreviations: HC, healthy controls; IS, sarcopenia only; OP, osteoporosis only; OS, osteosarcopenia; SM, sphingomyelin.

**Supplementary Table S3. Detailed statistical metrics of selected representative metabolites in the primary OP versus OS comparison.**

| Metabolite | VIP | P-Value | Q-Value | Fold Change | Trend |
| --- | --- | --- | --- | --- | --- |
| Leucine | 1.843 | 0.001 | 0.012 | 0.54 | ****↓**** |
| Hypoxanthine | 3.459 | 8.20E-06 | 0.010 | 0.41 | ****↓**** |
| Creatine | 2.313 | 2.00E-04 | 0.020 | 1.37 | ****↑**** |
| Sarcosine | 2.220 | 3.00E-04 | 0.021 | 1.22 | ****↑**** |
| Pyruvate | 1.237 | 0.001 | 0.029 | 1.27 | ****↑**** |
| SM(d34:1) | 1.859 | 0.003 | 0.046 | 2.12 | ****↑**** |
| SM(d18:1/17:0) | 2.11 | 0.001 | 0.042 | 0.79 | ****↓**** |
| Arachidonoylcarnitine (Car(20:4)) | 1.683 | 1.40E-05 | 0.016 | 0.62 | ****↓**** |
| 3-Methylglutaconic acid | 1.431 | 0.002 | 0.026 | 1.33 | ****↑**** |

Note: Fold change (FC) was calculated as the ratio of the median abundance in the OS group to that in the OP group. Arrows indicate the direction of change in OS relative to OP: ↑, upregulated; ↓, downregulated. The table presents the variable importance in projection (VIP) score, raw P-value, false discovery rate (FDR)-adjusted P-value (Q-value), fold change, and regulation trend for selected representative metabolites in the primary OP versus OS comparison. These metabolites were selected from FDR-significant metabolites based on their biological relevance to musculoskeletal-related pathways and their interpretability in the main text.

Abbreviations: OP, osteoporosis only; OS, osteosarcopenia; VIP, variable importance in projection; FDR, false discovery rate.

****Supplementary Note 1. Exogenous metabolites excluded from the exploratory overlap analysis.****

**In the exploratory analysis comparing metabolic overlaps between osteosarcopenia (OS), osteoporosis (OP), and isolated sarcopenia (IS) (Figure 3), 14 overlapping metabolites were initially identified. Seven of these were determined to be of exogenous origin and were therefore excluded from any further biological interpretation. These exogenous metabolites are listed below solely for transparency and completeness.**

**1. Tetradecyl sulfate (sodium)**

**2. [1,2,4] triazolo[4,3-b][1,2,4] triazole-3,7-diamine**

**3. 6-[2-(3-Hydroxy-4-methoxyphenyl)ethyl]-4-methoxy-5,6-dihydro-2H-pyran-2-one**

**4. alpha-Methylbutyrolactone**

**5. 14-(5-Ethyl-6-methylheptan-2-yl)-5-hydroxy-2,15-dimethyltetracyclo[8.7.0.0^{2,7}.0^{11,15}] heptadec-7-en-9-one**

**6. Benzamide, N-(4-amino-2-chlorophenyl)-5-chloro-2-hydroxy-**

**7. 1-(4-{[tert-Butyl(dimethyl)silyl] oxy}-6-fluoro-3,4-dihydro-2H-1-benzopyran-2-yl)-2-{[2-(6-fluoro-3,4-dihydro-2H-1-benzopyran-2-yl)-2-hydroxyethyl] amino}ethan-1-ol**

**Note: All seven endogenous metabolites from this overlap (Histidinol, Lactate, N6-Methyllysine, Car (18:0), 3-Aminopentanoic acid, SM(d34:1), and Heptadecanoyl carnitine) are listed in the main text (Results section). SM (d34:1) was the only one to meet full statistical criteria in both comparisons and was carried forward for further analysis.**

**Supplementary Table S4. Raw and covariate-adjusted P-values of selected representative metabolites in the primary OP versus OS comparison.**

| Metabolite | Raw P-value | Covariate-adjusted P-value | Trend |
| --- | --- | --- | --- |
| Leucine | 0.001 | 0.018 | ****↓**** |
| Hypoxanthine | 8.20E-06 | 3.32E-04 | ****↓**** |
| Creatine | 2.00E-04 | 1.70E-04 | ****↑**** |
| Sarcosine | 3.00E-04 | 2.25E-04 | ****↑**** |
| Pyruvate | 0.001 | 0.329 | ****↑**** |
| SM(d34:1) | 0.003 | 0.030 | ****↑**** |
| SM(d18:1/17:0) | 0.001 | 0.009 | ****↓**** |
| Arachidonoylcarnitine (Car(20:4)) | 1.40E-05 | 0.048 | ****↓**** |
| 3-Methylglutaconic acid | 0.002 | 0.020 | ****↑**** |

Note: Raw P-values were derived from the primary OP versus OS differential metabolite analysis. Adjusted P-values were derived from covariate-adjusted linear regression models restricted to OP and OS participants, with individual-level normalized metabolite abundance as the dependent variable and group status as the main independent variable, adjusting for age, sex, body mass index (BMI), and estimated glomerular filtration rate (eGFR). The adjusted P-value represents the P-value for the group term comparing OS with OP. Most selected representative metabolites remained nominally significant after covariate adjustment, whereas pyruvate was attenuated after adjustment. P-values are reported to three significant digits, with values smaller than 0.001 shown in scientific notation.

Abbreviations: OP, osteoporosis only; OS, osteosarcopenia;

**Supplementary Table S5. Performance of key metabolites in distinguishing osteosarcopenia (OS) from other groups via ROC curve analysis.**

| Comparison | Differential metabolite / model | AUC (95% CI) | Specificity | | Sensitivity |
| --- | --- | --- | --- | --- | --- |
| OP vs OS | Leucine | 0.79 (0.68–0.90) | | 0.70 | 0.90 |
|  | Sarcosine | 0.78 (0.65–0.90) | | 0.87 | 0.63 |
|  | **Hypoxanthine** | 0.87 (0.78–0.96) | | 0.80 | 0.90 |
|  | **Combined model** | 0.86 (0.76–0.96) | | 0.90 | 0.77 |
| HC vs OS | Isovalerylcarnitine | 0.82 (0.71–0.92) | | 0.70 | 0.86 |
|  | **Valerylcarnitine** | 0.82 (0.71–0.92) | | 0.70 | 0.86 |
|  | **SM(d34:1)** | 0.76 (0.64–0.89) | | 0.83 | 0.67 |

AUC values were calculated in the discovery cohort and were not externally validated; therefore, they should be interpreted as apparent discovery-set estimates rather than validated diagnostic performance.

Abbreviations: HC, healthy controls; IS, sarcopenia only; OP, osteoporosis only; OS, osteosarcopenia. HC vs OS: Group HC versus OS; OP vs OS: Group OP versus OS; AUC: area under the curve; CI: confidence interval.

**Supplementary Table S6. Annotation information for representative candidate metabolites discussed in the manuscript.**

| Metabolite | MS2 score | Annotation level | m/z | RT | Formula | Ion mode | HMDB ID | KEGG ID | **PubChem CID** |
| --- | --- | --- | --- | --- | --- | --- | --- | --- | --- |
| Leucine | 3.92 | Level 1 | 132.1016 | 169 | C6H13NO2 | POS | HMDB0000687 | C00123 | 6106 |
| Hypoxanthine | 3.95 | Level 1 | 135.0309 | 84.4 | C5H4N4O | NEG | HMDB0000157 | C00262 | 790 |
| Creatine | 3.92 | Level 1 | 132.0762 | 206.7 | C4H9N3O2 | POS | HMDB0000064 | C00300 | 586 |
| Sarcosine | 3.89 | Level 1 | 90.0547 | 206.7 | C3H7NO2 | POS | HMDB0000271 | C00213 | 1088 |
| SM(d18:1/17:0) | 3.89 | Level 1 | 717.5878 | 119.3 | C40H81N2O6P | POS | HMDB0240609 | NA | 46891763 |
| Arachidonoylcarnitine / Car(20:4) | 3.85 | Level 1 | 448.3408 | 109.4 | C27H45NO4 | POS | HMDB0006455 | NA | 136212424 |
| Pyruvate | 3.91 | Level 1 | 87.0086 | 52.5 | C3H4O3 | NEG | HMDB0000243 | C00022 | 1060 |
| Proline | 3.93 | Level 1 | 116.0702 | 187.3 | C5H9NO2 | POS | HMDB0000162 | C00148 | 145742 |
| Isovalerylcarnitine / Car(5:0) | 3.90 | Level 1 | 246.1695 | 151.5 | C12H23NO4 | POS | HMDB0000688 | C20826 | 169235 |
| Valerylcarnitine | 3.92 | Level 1 | 246.1695 | 151.5 | C12H23NO4 | POS | HMDB0013128 | NA | 16226475 |
| SM(d34:1) | 2.68 | Level 2 | 703.572 | 28.9 | C39H79N2O6P | POS | NA | NA | NA |
| 3-Methylglutaconic acid | 2.71 | Level 2 | 143.0346 | 208.3 | C6H8O4 | NEG | HMDB0000522 | NA | 1551553 |

Note: This table provides annotation information only for representative candidate metabolites highlighted or discussed in the manuscript, rather than for all annotated metabolites in the dataset. Level 1 annotation was defined as matching with authentic standards using retention time (RT), MS1, and MS2 information. Level 2 annotation was defined as putative annotation based on MS1/MS2 spectral matching against spectral databases. NA indicates that the corresponding database identifier was not available or not assigned. POS, positive ionization mode; NEG, negative ionization mode.
